# Effect of landscape design on depth perception in classical Chinese gardens: A quantitative analysis using virtual reality simulation

**DOI:** 10.3389/fpsyg.2022.963600

**Published:** 2022-11-03

**Authors:** Haipeng Zhu, Zongchao Gu, Ryuzo Ohno, Yuhang Kong

**Affiliations:** ^1^Department of Architecture, School of Architecture, Tianjin University, Tianjin, China; ^2^School of Architecture and Fine Art, Dalian University of Technology, Dalian, China; ^3^Tokyo Institute of Technology, Tokyo, Japan

**Keywords:** landscape perception, design, depth perception, visual depth cues, VR experiment

## Abstract

It is common for visitors to have rich and varied experiences in the limited space of a classical Chinese garden. This leads to the sense that the garden’s scale is much larger than it really is. A main reason for this perceptual bias is the gardener’s manipulation of visual information. Most studies have discussed this phenomenon in terms of qualitative description with fragmented perspectives taken from static points, without considering ambient visual information or continuously changing observation points. A general question arises, then, on why depth perception can vary from one observation point to another along a garden path. To better understand the spatial experience in classical Chinese gardens, this study focused on variations in perceived depth among different observation points and aimed to identify influential visual information through psychophysical experimentation. As stimuli for the experiment, panoramic photos of Liu garden were taken from three positions at Lvyin Pavilion. Considering the effects of pictorial visual cues on depth perception, the photos were processed to create 18 kinds of stimuli (six image treatments * three positions). Two tasks were presented to the participants. In Task 1, 71 participants were asked to rate the depth value of the garden using the magnitude estimation method in a cave automatic virtual environment (CAVE). Statistical analysis of Task 1 revealed that depth values differed significantly among different viewpoints. In Task 2, participants were asked to compare 18 stimuli and 3D images presented on three connected monitors and to judge the depth of the garden using the adjustment method. The results of Task 2 again showed that depth values differed significantly among different viewpoints. In both tasks, ambient information (i.e., the perspective of interior space) significantly influenced depth perception.

## Introduction

Landscape design has long been known to strongly affect residents’ sense of well-being (Matsuoka and Kaplan, 2008; Cottet et al., 2018). Understanding user perception is a useful tool in landscape design (Rey Gozalo et al., 2019). Previous studies have explored the relationship between environmental information and subjective perception in landscape design, including the characteristics of environmental components, such as plant size, texture, and color (Serpa and Muhar, 1996; Cary and Williams, 2002; Kaufman and Lohr, 2004; Kendal et al., 2012; Wang and Zhao, 2020), the complexity and structure of vegetation (van den Berg and van Winsum-Westra, 2010) and the design intensity of artificiality (Xu et al., 2018) can shape user’s spatial perception and affect esthetic preference.

Depth, or distance, perception is one of the important aspects of spatial perception. Distance estimation and judgment have been conducted in small-scale environments (Crompton and Brown, 2006), in large-scale natural environments (Okabe et al., 1986), and in cities (Canter and Tagg, 1975). Depth/distance perception is affected by spatial features, including such pathway design features as spaciousness, numbers of turns, brightness (Ohno et al., 2006), space width and height (Ohno et al., 2001), slope, and winding trails (Okabe et al., 1986). The previous studies showed that the perceived distance plays an important role in perceiving and understanding environments, which was as a function of direction (Lee, 1970) and the function of time (Crompton and Brown, 2006) in a city. Studies of distance perception have revealed potential function on landscape design, which motivates our focus on the influence of perceived distance on landscape design.

Typical phenomena in visiting a classical Chinese garden include uncertainty of depth judgment, distances that seem deeper than the actual distance, and perceptual bias as visitors move over short distances. The manipulation of depth, an essential parameter for judging a garden’s scale, enables visitors to feel the garden is larger than it really is while enjoying a rich experience. Existing studies have described the influence of landscape design on the perception of spatial depth from two aspects, mainly from a qualitative perspective. On the one hand, the uncertainty of garden depth perception and the perception of deeper garden depth during the garden tour is pointed out from the perspective of physical experience (Fung et al., 2016; Tong, 2016); on the other hand, the possible influence of the organization of environmental components on depth perception is pointed out from the perspective of gardening techniques (Li and Jan, 2009; Li, 2021). To provide a more precise and comprehensive understanding of the perceptual characteristics of classical Chinese gardens. This study further quantitatively verifies the relationship between depth perception and visual information based on existing qualitative studies.

This study addressed the following research questions. Does depth perception change at close interval positions? Do changes in visual information caused by different viewpoints affect depth perception? Finally, what is the relationship between visual information and depth perception?

Our experiment aimed to assess the depth value of the garden as the viewpoint changed at a short distance (one or two human steps). Our first hypothesis (H1), which concerns the effect of observer’s environment, was as follows: there will be significant differences in participants’ depth perception among small point-of-view displacements which provide different composition of the ambient visual information framing the garden landscape.

Based on previous studies noting that depth cues influence depth perception, this aspect was systematically varied in the two experimental tasks. Thus, our second hypothesis (H2), which concerns the effect of the layout of elements in the garden, was as follows: variation in visual information (pictorial depth cues: perspective, texture gradient, and occlusion) will affect (enhance or reduce) participants’ perceived depth of garden.

## Related works

### Basics of visual depth perception

Depth perception discussed in the present study means egocentric distance perception, that is, the subjectively perceived distance from an observer to an object (Renner et al., 2013). Visual depth cues include pictorial, motion-induced, and structural cues (Watson and Enns, 2012; Maruhn et al., 2019). Pictorial depth cues are derived from characteristics and features of a two-dimensional image (Renner et al., 2013), such as occlusion, relative size, relative height, familiar size, linear perspective, texture gradient, atmospheric perspective, and shadowing (Schwartz and Krantz, 2017). Motion-induced visual cues are changes in visual information caused by the motion of observers or objects (Maruhn et al., 2019), such as motion parallax, deletion and accretion, and optic flow (Schwartz and Krantz, 2017). Structural depth cues refer to physical adjustments and anatomic relations between the two human eyes, including stereopsis, accommodation, and vergence (Watson and Enns, 2012).

Considering the panoramic images used in the present research, we focused on the effects of pictorial depth cues. Combining the cases used in the experiment, we investigated mainly occlusion, linear perspective, and texture gradient. Occlusion occurs when an object partially obstructs the view of a second object. We infer that the hidden object is farther away from us than the obstructing object (Schwartz and Krantz, 2017). Linear perspective is a pictorial depth cue that arises from parallel lines appearing to converge as they recede into the distance (Schwartz and Krantz, 2017). Texture gradient is a monocular depth cue that arises because the texture becomes finer as it recedes into the distance; texture gradients are clearly related to relative size (Schwartz and Krantz, 2017). Gibson (1951) first noted the effect of ground texture on distance perception.

In addition to depth cues, distance perception is influenced by environmental context. Lappin et al. (2006) conducted two experiments in a lobby, a hallway, and an open lawn. The results show that the accuracy of perceived distance is affected by the surrounding environment. Witt et al. (2007) investigated perceived distance in two types of environments (indoors and outdoors) with constant depth-related variables. The derived differences in perceived distance show the influence of variations in space. Thus, these studies show that environmental context can influence distance perception.

### Methods for investigating depth perception

Distance estimation methods are classified into three categories: verbal estimates, perceptual matching, and visually directed actions (Montello, 1991; Renner et al., 2013). In verbal estimates, participants determine the distance between themselves and an object based on a unit of measurement (Loomis and Knapp, 2003). This method is convenient and commonly used, as it is easy to obtain the participant’s distance estimate (Renner et al., 2013), but the results are influenced by not only perception but also cognitive factors, e.g., knowledge and deductive reasoning (Loomis and Knapp, 2003). In perceptual matching tasks, participants are asked to adjust the position of a marker to an equivalent distance from an object in one direction (Renner et al., 2013). This method is thought to be less influenced by cognitive factors (Loomis and Philbeck, 2008). A variant of perceptual matching is bisection, where participants indicate the midpoint of an egocentric distance (Renner et al., 2013). The relative distance provided in bisection tasks improves measurement accuracy (Rieser et al., 1990). In visually directed actions, the participant views the distance to the target object, then is blindfolded and performs an action toward the target object (Renner et al., 2013). The most common of these measurement actions include blind walking, triangulated blind walking, and time imagined walking (Renner et al., 2013).

For depth measurement, magnitude estimation and adjustment are used in this research; these methods comprise verbal estimates and perceptual matching, respectively. In magnitude estimation, participants estimate the magnitude of physical stimuli by assigning numerical values proportional to the stimulus magnitude they perceive (Stevens, 1975). This method usually proceeds as follows. A standard stimulus is presented as a modulus, which is 100. Then, the participant must judge subsequent stimuli and give a numerical value comparing them to the modulus. Environmental studies have used magnitude estimation to measure the depth and distance perception of underground paths (Ohno et al., 2006). In the adjustment method, a participant adjusts a variable stimulus to match a constant or standard. For example, the observer is shown a standard visual stimulus of a specific intensity and is asked to adjust a comparison stimulus to match the brightness of the standard. This method has been used to measure multiple sensations, such as color and sound perception (Mollon, 1992).

### Application of panoramic images in psychological research

Photographic simulation has been used widely to determine user visual perception and valuation (Winkel et al., 1970; Shuttleworth, 1980; Nausser, 1982; Law and Zube, 1983; Zube et al., 1987). Panoramic images offer certain advantages as an approach to collecting and recording environmental information. Compared with traditional photographs, panoramic images allow for recording ambient information surrounding an observation point. Thiel (1997) suggest that hemispherical projection can be used as a two-dimensional graphic to provide a comprehensive, metrical mapping of environments from an ego-centered perspective. Panoramic images provide a broader area and a quick global impression, integrating the visible surfaces of various components and the distances between surfaces and the observation point (Ohno, 1993). Further, environmental surfaces are often statistically analyzed based on panoramic projections (Pardo-García and Mérida-Rodríguez, 2017). Examples include determining green vegetation indexes (coverage of urban surfaces by tree crowns) based on Google Street View panoramas (Li et al., 2015; Liu et al., 2019).

Panoramic images provide valid environmental representations for psychological research. Various studies have investigated the hypothesis that spatial evaluation of indoor and exterior spaces using the whole sky image is better than using conventional panoramic images as an alternative to real spaces. Similarities have been found between the results obtained from on-site experiments and whole sky image experiments (Akiyama et al., 2018; Sugita et al., 2018). The 360° panoramas taken from real environments were digitally processed to create immersive virtual environmental stimuli that depict variations of characteristics of vegetation (Tabrizian et al., 2018). Empirical studies presenting panoramic images as stimuli have provided all directions surrounding the perceiver, thereby estimating environmental perception in relation to integrated visual information. Inagami (2015) used this approach to examine the relationship between complexity and esthetic feelings in Japanese gardens. The results show that interest increases with complexity, but beauty and preference reach a plateau asymptotically.

## Research methods

This study examined the relationship between depth perception and visual information based on two approaches: different experimental set-ups and different methods of obtaining perceptual responses. These approaches are expected to provide a more comprehensive understanding of the perceptual characteristics of classical Chinese gardens.

### Study area

Liu Garden served as our research case. This is a classical Chinese garden established in 1593, located in Suzhou, China. To examine the effect of ambient visual information on depth perception, the panoramas were taken from inside of the Lvyin Pavilion, which was designed as a station point from which the entire central part of the garden could be viewed. The ambient information considered is composed of the frame formed by the walls, ceiling and handrails, as well as the interior space of the pavilion. Qualitative studies have shown that people have uncertain judgments of the spatial depth of the garden here (Fung et al., 2016; Tong, 2016). The location of the internal observation point was chosen mainly in consideration of the degree of variability of environmental components between positions. As the width of the Lvyin Pavilion is small (4.5 m * 3.5 m), the three viewing points were chosen to provide a distinctly different composition of the ambient visual information framing the garden landscape. The panoramas were taken horizontally from the left, middle, and right position in Lvyin Pavilion. The interval distance among the three positions was 1.5 m (one human step = 0.5 m).

### Stimuli: Processed panoramic images

**Figure 1:**
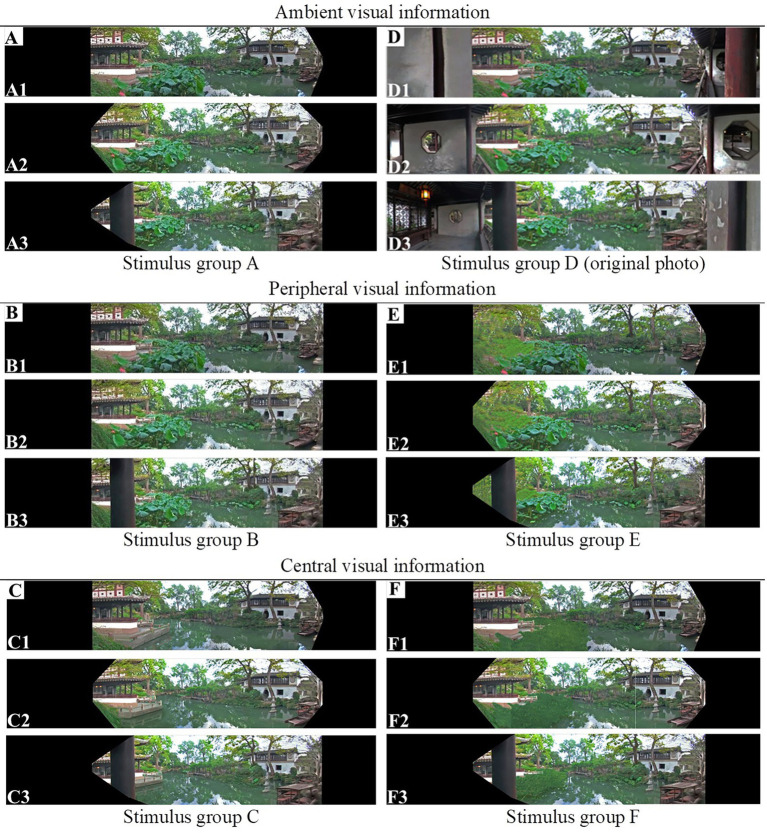
Stimulus groups of panoramic images with different depth cues: (**A** and **D**) Ambient visual information; (**B** and **E**) Peripheral visual information; (**C** and **F**) Central visual information. (In each group, the pictures taken from the left, middle, and right positions are shown from top to bottom.)

To examine the effect of pictorial depth cues on depth perception, the panoramas were processed based on three original photos to create 18 kinds of stimuli (six image treatments * three positions), as shown in Table 1; Figure 1. Each stimulus group was intended to control different depth clues: stimulus group A was processed to darken the interior space of the pavilion to remove the linear perspective effect of the space; stimulus group B was processed to make the peripheral frame rectangular to remove the effect of the frame shape; stimulus group C was processed to replace the overlapped image of lotus leaves with water to remove the occlusion effect; stimulus group D was unchanged from the original panorama, retaining all depth cues; stimulus group E was processed to erase the waterside buildings to remove the linear perspective effect; and stimulus group F was processed to blur the texture of the lotus leaves to remove the texture gradient effect. Each stimulus group was also characterized by the category of visual information considered: stimulus groups C and F were concerned with central visual information; stimulus groups B and E were concerned with peripheral information; and stimulus groups A and D were concerned with ambient border information.

**Table 1:** The 18 kinds of stimuli (six image treatments * three positions).

		Stimulus group		Position (left, middle, right)
Original panorama		D	Retain all depth cues	(D1, D2, D3)
Treatment of depth cue	Occlusion	B	Make peripheral frame rectangular to remove the effect of frame shape	(B1, B2, B3)
		C	Replace overlapped image of lotus leaves with water to remove their occlusion effect	(C1, C2, C3)
	Linear perspective	A	Darken interior space of the pavilion to remove the linear perspective effect of the space	(A1, A2, A3)
		E	Erase waterside buildings to remove the linear perspective effect	(E1, E2, E3)
	Texture gradient	F	Blur texture of the lotus leaves to remove the texture gradient effect	(F1, F2, F3)

The garden scene, as a target of focal vision, used in this experiment is quite far from the observer (further than 10 m), the binocular cues caused by convergence are not significant due to the small angle (Okoshi, 2012), while ambient visual information from the pavilion space is not relevant to binocular cues. The effects of binocular cues are not considered in this study.

### Relative 3D models

**Figure 2:**
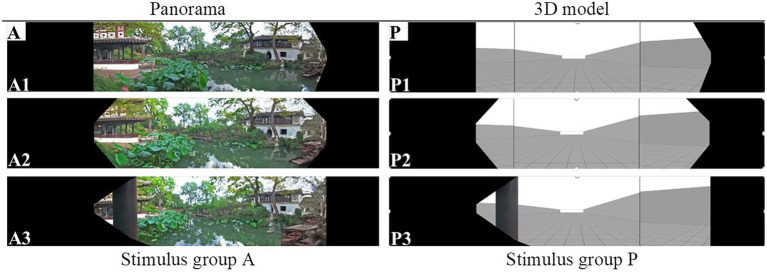
Example stimuli: panoramas and corresponding 3D models. (In each group, the pictures taken from the left, middle, and right positions are shown from top to bottom.)

For Experiment 2, three-dimensional (3D) models were built to simulate the real situation corresponding to the three positions of the panoramas: relative position (to provide a similar perspective), grids on the ground floor (1.5 m × 1.5 m to provide depth cues to support judging distance), and boundaries of the garden (to restrict horizontal width and manipulate the front wall to control depth). Virtual models were presented on three connected monitors in Task 2. Each monitor had a16:9 aspect ratio, which is the same as the projection wall of the CAVE (cave automatic virtual environment) used in Task 1. The angle between adjacent screens was modified to avoid distorting the image and to achieve a natural view from the participant’s perspective. Figure 2 provides a comparative example view between the panoramic images and 3D models.

### General design of experiment

Five sessions were conducted in the two experiments (Table 2). The magnitude estimation and adjustment methods were used to study the effect of the variables (i.e., position and depth cues) on depth perception. Table 3 provides the overview of the settings in the two tasks.

**Table 2:** The five sessions of the experiment.

Session	Stimulus group	Depth cue	Category of visual information
1	A and B	Frames formed by walls, ceiling, and handrail	Peripheral information
2	A and C	Image of lotus leaves overlapping water	Central information
3	A and D	Interior space of pavilion	Ambient information
4	A and E	Waterside buildings	Peripheral information
5	A and F	Texture of lotus leaves	Central information

**Table 3:** General overview of the two tasks.

		Task 1		Task 2	
		Controlled group	Experimental group	Controlled group	Experimental group
Participants		71	13–16	71	13–16
Stimuli	Standard	A2	A2	Group A (A1, A2, A3)	Groups B-F (B1, B2, B3, C1, …, F3)
	Comparative	Group A (A1, A3)	Groups B-F (B1, B2, B3, C1, …, F3)	3D model P (P1, P2, P3)	3D model P (P1, P2, P3)
Subjective response		Depth value (ME value)		Estimated distance (m)	
Experimental setup		Cave automatic virtual environment		Three connected monitors	
Method to obtain participant response		Method of Magnitude Estimation		Method of Adjustment	

**Figure 3:**
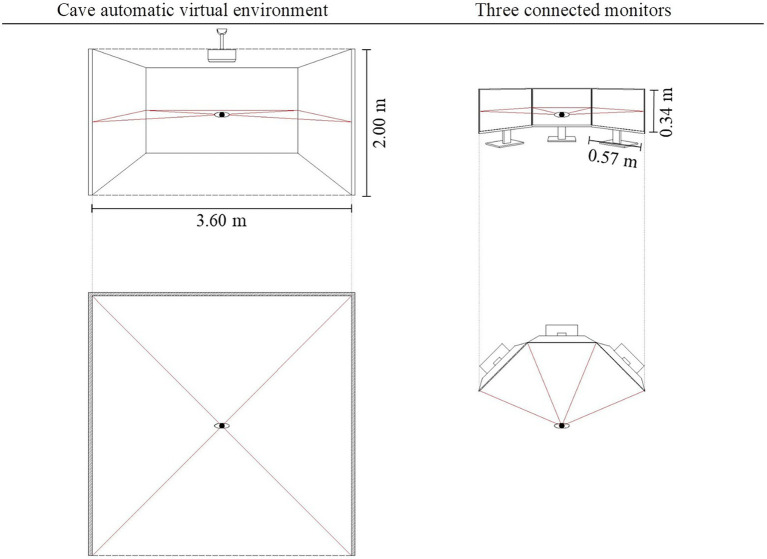
Schematic diagram of the experimental setup and representation display.

In the two tasks, participants evaluated depth values using psychophysical methods under different experimental circumstances. The main differences between the two tasks were twofold. First, the experimental setup was different in each, as shown in Figure 3. In Task 1, participants experienced scenes in a Cave Automatic Virtual Environment (CAVE), an immersive virtual reality environment where projectors are directed to between three and six of the walls of a room-sized cube. Compare to the Head-mounted VR, CAVE can provide central and ambient visual information at the same time. The detail of experiment environment setting was given in Section “Method”. In Task 2, panoramas were shown in a conventional way using three connected monitors. Second, the methods used to obtain participant responses concerning depth perception differed. In Task 1, the magnitude estimation method was used to obtain depth values based on the general impression and atmosphere of the stimuli. In Task 2, by carefully comparing two stimuli, participants tried to adjust the 3D model to a relative position to obtain a similar depth feeling.

## Task 1

### Method

Participants. A total of 71 participants (23 men and 48 women) were recruited. Their ages ranged from 19 to 39 years (*M* = 23.8, SD = 4.1). Participants were recruited *via* the participant recruitment platform. Participants were required to have normal or corrected-to-normal vision (with contact lenses) to participate. None of the participants had visited Liu Garden or had professional experience in architecture, landscape design, or related fields.

Materials. The experiment was conducted in the virtual reality lab. It has a CAVE with three large projection walls (3.6 m * 2.0 m), which provided an immersive experience for participants. A seat was placed at the center of the CAVE at a distance of 1.8 m from the front facing the projection wall. We adjusted the stimulus picture projection according to the location of the laboratory viewpoint and screen to ensure that the source direction of the visual information was consistent with the real environment. The CAVE setup as above is designed to ensure that participants experience the same ambient optical array, size and relative orientation of the scenery as in the real world. The lights were turned off during the experiment.

The experiment consisted of five sessions. Each session was designed to test the effects of observation position and visual depth cues on participant depth perception. Participants were divided into five groups. In each session, stimulus A2 (see Figure 2) served as the standard stimulus (depth value: 100). The comparative stimuli included stimulus group A in the other position (A1, A3) and one of the stimulus groups. For example, session 1 included stimulus groups A (A1 and A3) and B (B1, B2, and B3) as comparative stimuli, which were rated and assigned a number by comparing the standard stimulus A2.

Procedure. Upon arrival, participants were given an information sheet that described the experiment and were asked to sign the consent form. Participants were then asked to sit on the chair at the center of the CAVE.

**Figure 4:**
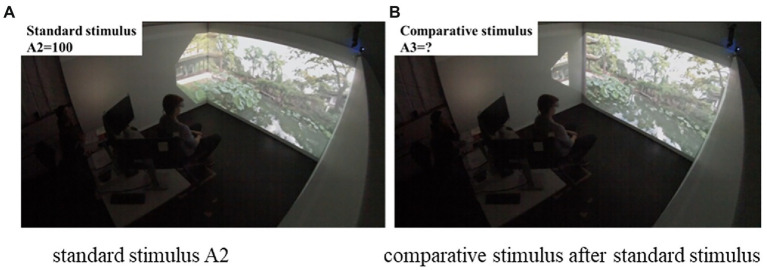
A student participating in Task 1: **(A)** Standard stimulus A2; **(B)** Comparative stimulus after standard stimulus.

For each participant, a test trial was conducted first to help familiarize participants with the process and the definition of depth in this study (i.e., the physical distance from the participant’s position to the end of the garden). Task 1 contained five trials. For each, the participant first viewed the standard stimulus A2 with a depth value of 100 for 45 s. After that, the participant experienced a comparative stimulus for 45 s and assigned a depth value based on the impression of the standard stimulus. The comparative stimuli were tested in random order. Participants were asked to close their eyes for 15 s before the next trial started (Figure 4).

### Results

Among the data obtained from all participants (*N* = 71), seven participants’ data were removed because of outliers and incorrect operation. Outlier detection was performed on the depth values using IBM SPSS 20.0. Incorrect operations detected during the experiment included misunderstanding the definition of depth and overestimating depth value.

#### Differences in depth perception among observation points (H1)

**Figure 5:**
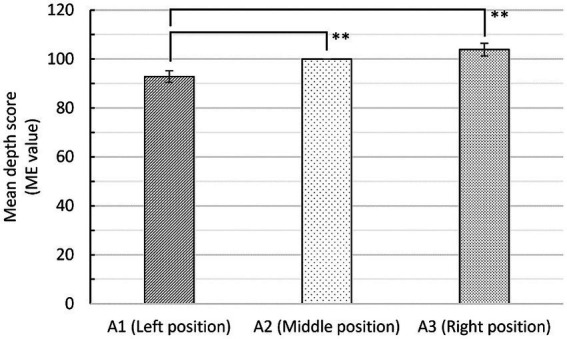
Mean depth scores (ME value) (± standard error) in three positions in Task 1. ^**^Significance (*p* < 0.01).

Figure 5 shows the mean depth scores in three positions of stimulus group A (*N* = 64, *M* = 23.41, SD = 3.61). The depth scores were evaluated using magnitude estimation (i.e., ME value). The mean depth scores (ME value) of the left and right positions were 92.86 (SD = 19.11) and 103.84 (SD = 20.74), respectively. The middle position was treated as a standard value of 100. A one-sample *t*-test revealed significant differences in mean depth scores between the left and middle positions, 95% CI [−11.91 to −2.37], *t* (63) = −2.989, *p* = 0.004, *d* = 0.528. A paired-sample *t*-test showed significant differences between the left and right positions, 95% CI [−18.90 to −3.07], *t* (63) = −2.774, *p* = 0.007, *d* = 0.551. This clearly shows that the panorama taken in the right position was perceived as much deeper than the left position.

#### Effects of visual cues on depth perception (H2)

Average depth values among the three positions were calculated for five sessions with ambient, central, and peripheral information (Table 4). In session 3, the average depth of stimulus group D (pictures with the interior space) was higher than that of group A. A paired-sample *t*-test showed no significant difference between the average depth values of stimulus groups A and B, A and C, A and E, and A and F, except in session 3 (i.e., A and D; *t* (11) = −2.798, *p* = 0.017, *d* = 0.942). This means that the ambient information (i.e., the pavilion’s interior space) significantly influenced depth perception.

**Table 4:** Average depth scores among the three positions in each session in Task 1.

Visual information	Session	Stimulus group	*N*	*M* (ME value)	Sig. (two-tailed)	95% Confidence Interval
						Lower	Upper
Ambient information	3	A	12	102.50	0.017*	−24.81	−2.96
		D		116.39			
Central information	2	A	13	99.87	0.740	−11.97	8.74
		C		101.49			
	5	A	13	96.03	0.990	−8.49	8.39
		F		96.08			
Peripheral information	1	A	12	97.89	0.663	−11.17	7.39
		B		99.78			
	4	A	14	98.45	0.801	−10.06	12.77
		E		97.10			

* Significance (*p* < 0.05).

**Figure 6:**
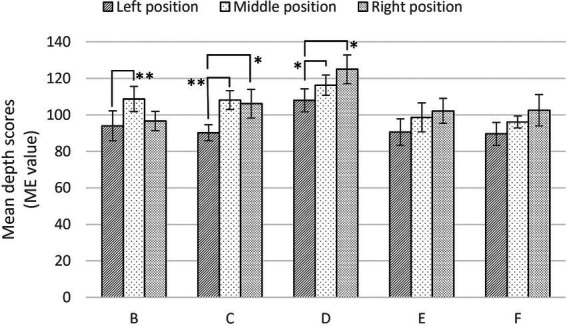
Mean depth scores (± standard error) with different visual cues in Task 1. ^*^Significance, (*p* < 0.05). ^**^Significance (*p* < 0.01).

Regarding stimulus groups B through F, which were processed to have different visual cues, Figure 6 shows the mean depth scores of the three observation points. In stimulus groups B and C, the participants sensed the most depth in the middle position instead of the right position. This means that the treatment of depth cues (i.e., removing the effect of frame shape and overlapping lotus leaves on water) affected the general trend of depth value; specifically, participants perceived much more depth in the right position than the left position. In stimulus groups E and F, there were no significant differences among the three positions, highlighting the potential effects of depth cues (i.e., linear perspective of waterside buildings and texture of lotus leaves) on depth perception.

### Discussion

Generally, Task 1 showed that as participants gradually changed their observation points from a static viewpoint (e.g., the pavilion), depth perception changed significantly. Surprisingly, compared to the central and peripheral information, ambient information (i.e., the perspective of the pavilion’s interior space) significantly affected depth perception. Further, the visual cues had varying degrees of impact on depth perception. For example, after removing the effect of the frame shape and the overlap of lotus leaves on water, participants perceived the most depth in the middle position rather than the right position. Removing the linear perspective of the waterside buildings and the texture of the lotus leaves revealed the potential effect on depth perception; however, further research is needed due to insufficient sample size.

## Task 2

### Method

#### Participants

Seventy-one participants were assigned to five sessions in Task 2. Depth value was re-estimated in different experimental circumstances using the adjustment method.

#### Materials

Task 2 was conducted using three connected monitors that represented the continuous image at static angles. A seat was placed in front of the desk facing the midpoint of the three monitors. The height of the chair could be adjusted to keep the participant’s eyesight focused on the middle level of the monitors. The lights were turned off during the experiment.

The five sessions were re-tested in Task 2. In each session, group P (P1, P2, and P3), a relative 3D model of the left, middle, and right positions, was manipulated to match the depth perceived from the panorama in the corresponding position. For example, panorama groups A (A1, A2, and A3) and B (B1, B2, and B3) were tested in random order in session 1.

#### Procedure

**Figure 7:**
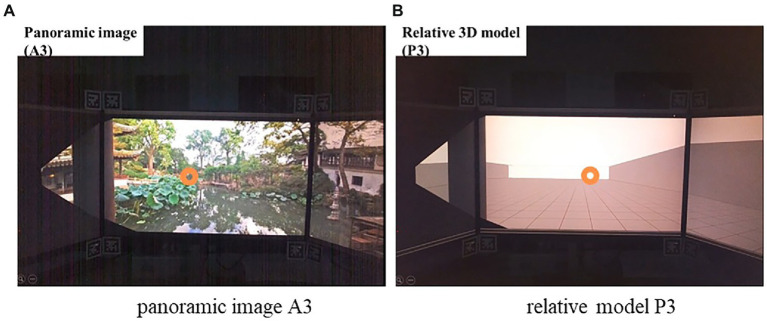
A student participating in Task 2 (orange circle: gaze point): **(A)** Panoramic image A3; **(B)** Relative model P3.

A test trial was conducted to familiarize participants with the method used in Task 2. Our definition of depth is the distance from the participant’s position to the end of the garden. Here, the end of the garden was determined by the position of the target wall in the 3D model. The starting position of the target wall was 200 m in the relative model. The real distance of the garden is 60 m. The experiment contained six trials. In each trial, two stimuli were presented to participants: the panorama and its relative model (e.g., stimulus A1 and stimulus P1). The participant was asked to use the “Alt + Esc” buttons on the keyboard to switch stimuli for comparison and the “Up + Down” buttons to adjust the target wall in the 3D model. The participant could adjust as many times as desired before matching the depth perceived from the panorama. The panoramas were presented in random order. A blank picture (gray color) was inserted between the panorama and the 3D model to avoid afterimage influence (Figure 7).

### Results

Participants’ estimated distance was calculated from the position of the target wall in the 3D model. Using SPSS, no outliers were found in the 71 participants’ data. However, almost all results showed that the perceived distance was about twice as high as the actual distance from the viewpoint to the target wall in the garden. Although overestimation of distance was a consistent trend (which may have been caused by the difference in the amount of visual information between the panorama and the 3D model), we considered that the relative relationship of the results was maintained and worthy of analysis.

#### Differences in depth perception among observation points (H1)

**Figure 8:**
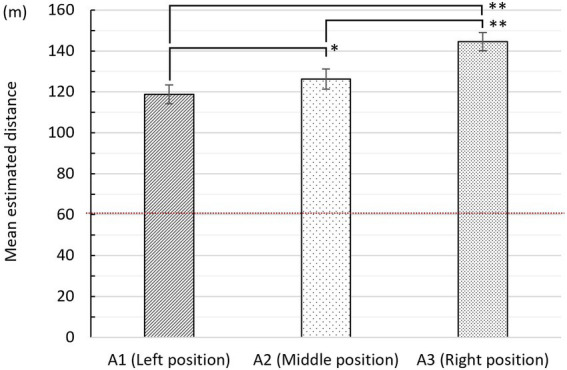
Mean estimated distance (± standard error) for the three positions in Experiment 2. ^*^Significance, (*p* < 0.05). ^**^Significance (*p* < 0.01); the actual depth of the garden is 60  m.

Figure 8 presents the mean estimated distance obtained using the adjustment method (group A, *N* = 71). The mean estimated distances for the left, middle, and right positions were 118.79 m (SD = 38.90), 126.28 m (SD = 42.09), and 144.54 m (SD = 37.23), respectively. Generally, as the observation points changed from left to right, longer distances were perceived. A repeated measures ANOVA with a Greenhouse–Geisser correction determined that mean depth value differed statistically among the three positions (*F* (1.900, 133.027) = 25.725, *p* < 0.001, *d* = 0.857). A paired-sample *t*-test revealed significant differences in depth perception between the left and middle positions, 95% CI [−14.15 to −0.84], *t* (70) = −2.246, *p* = 0.028, *d* = 0.185, middle and right positions, 95% CI [−25.52 to −10.97], *t* (70) = −5.009, *p* < 0.001, *d* = 0.460, and left and right positions, 95% CI [−33.85 to −17.65], *t* (70) = −6.339, *p* < 0.001, *d* = 0.676. Thus, H1 is supported.

#### Effect of visual cues on depth perception (H2)

Table 5 shows results for average estimated distance among the three positions in each session. The estimated distances for all experimental groups showed varying degrees of increase. However, a paired-sample *t*-test showed no significant differences in all sessions, except for session 3 (i.e., A vs. D; *t* (14) = −3.099, *p* = 0.008, *d* = 0.325). This means Task 2 further demonstrated the effect of interior space on depth perception.

**Table 5:** Average estimated distance among the three positions in each session in Task 2.

Visual information	Session	Stimulus group	*N*	*M (SD)* (m)	Sig. (two-tailed)	95% Confidence interval
						Lower	Upper
Ambient information	3	A	15	137.91	0.008**	−18.95	−3.45
		D		149.11			
Central information	2	A	13	128.51	0.135	−22.62	3.44
		C		138.10			
	5	A	16	120.46	0.232	−13.10	3.43
		F		125.29			
Peripheral information	1	A	14	126.82	0.404	−16.43	7.10
		B		131.49			
	4	A	14	136.10	0.547	−13.49	−7.49
		E		139.10			

** Significance (*p* < 0.01).

**Figure 9:**
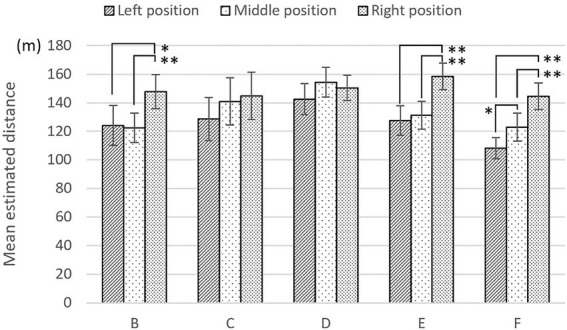
Mean estimated distance (± standard error) with different visual information in Task 2. ^*^Significance, (*p* < 0.05). ^**^Significance (*p* < 0.01).

Figure 9 shows mean estimated distances for stimulus groups B through F. In stimulus group C, there were no significant differences among the three positions. This potentially reveals the occlusion effect of the image of lotus leaves overlapping water on depth perception. Considering the limitations of sample size, future work could further investigate this issue.

### Discussion

The results of Task 2 further support the hypotheses 1 and 2 examined in Task 1. Importantly, we found that the significant differences in depth values among the three positions and the perspective of the pavilion’s interior space significantly influenced depth perception. Average depth values were elevated in stimulus groups B (pictures with peripheral frame removed) and C (pictures with lotus leaves removed), but no significant differences were observed. In addition, this shows the potential effect of lotus leaves overlapping water on depth perception; future work could further explore this issue.

## General discussion

Two tasks were used to test participant depth perception from different observation points at close intervals under controlled laboratory conditions. Comparing the results of the two tasks provides several interesting insights:

First, significant differences were found in depth values among the observation points (H1).

Second, ambient information (i.e., the perspective of the interior space) significantly affected depth perception (H2).

Third, compared with Task 2, depth perception in Task 1 was more strongly correlated with a wider range of variables. This was possibly related to the differences in the experimental setups and the perceptual measurements used in the two tasks.

### The garden changes its aspect at every turn

This research quantitatively demonstrates the phenomenon in classical Chinese gardens, whereby the garden changes its aspect at every turn. This study reveals this phenomenon from a static viewpoint (e.g., the pavilion) as the observation points change at close interval. The results show significant differences in depth values among positions using both magnitude estimation and the adjustment method (Tasks 1 and 2, respectively). Most research on this phenomenon of classical Chinese gardens has been based on qualitative descriptions of static and fixed positions (Fung, 2015). We further quantitatively measured subjective perception from several perspectives (at a close interval) and attempted to investigate the effective pictorial depth cues. This can be seen as one step toward an empirical understanding of the spatial experience in classical Chinese gardens.

Environmental perception is a dynamic process, where “perceiving is more likely to be a continuous, dynamic, ‘online’ (immediate) process” (Heft and Nasar, 2000). People can better perceive and understand environments in the context of sequential experiences; for example, moving through a landscape, as opposed to having a static perspective, can enhance spatial experiences (Thiel, 1964; Gibson, 2014). Further, Thiel (1997) holds that environmental design can thus be understood *via* the eye-level experience of users in the course of their movement through the environment. Various studies have calculated the visual information perceived from observation points along a route, for example, visible area of environmental components (Ohno and Kondo, 1994), and average horizontal distance to surrounding surfaces (Inagami and Ohno, 2010). Heft and Nasar (2000) integrated people’s responses to motion-induced and pictorial cues *via* dynamic and static displays. The results indicated different perceptual characteristics between them. Future work will further develop the impacts of motion-induced cues on depth/distance perception in classical Chinese gardens in a sequential context.

### Effects of ambient information on visual attention

In both tasks, ambient visual information unexpectedly affected depth perception compared with central and peripheral information. That is, stimulus group D (panoramas with the pavilion’s interior space) provided broader visual information. First, our results highlight the potential limitations of environmental perception studies based on ordinary pictures. Compared with panoramic images, partial pictures capture visual information from a limited area facing the front, regardless of the surrounding environment. In contrast, our approach is more in line with the way people obtain visual information in the real environment, that is, not just from a limited visual field, but freely viewing the surrounding information of the visual world (Gibson, 1951; Thiel, 1997). This reveals the advantage of panoramas is in recording the visible surface in the 360° range centered from an observation point, which also provides an analytical medium for quantifying environmental information. Inagami et al. (2008) investigated the correlation between the feeling of oppression and the visible area of environmental factors using four different widths of view angle, ranging from a limited view in front to a full 360° view. The results show that the feeling of oppression has the highest correlation with the 360° view. Barton et al. (2014) examined the effect of visual field on navigation performance and found the larger size of the visual field lead to an efficient route choice. Dupont et al. (2014) tested the effect of the landscape characteristics and photograph types on the observation pattern. The results show that panoramic and detail photographs are observed differently than the other types.

We attempted to interpret similarity in the results with two modes of vision, namely, focal and ambient vision. Ohno (2000) proposed a hypothetical model of environmental perception based on two theories: one, a two parallel processing vision system (Bassi, 1989), and the other, Gibson’s ambient optic array filling the visual environment (Gibson, 2014). Focal vision is used to eliminate unnecessary information from the surrounding environment *via* selective attention, while enhancing the detailed inspection and recognition of the target object (Ohno, 2000). Ambient vision is used to provide rapid global impressions or sensations without consciousness based on limited information from the broad environment (Ohno, 2000).

Regarding differences in the experimental set-ups, distance perception was more correlated with ambient vision in Task 1, while focal vision was more effective in Task 2. In Task 1, estimation of the subject’s sensation depended on the ambient atmosphere and the approximate feeling in the real environment provided by the immersive CAVE experience. Participants unconsciously picked up the visual information and generated a global impression or feeling. In stimulus group D, the emerging ambient information enhanced depth perception. In Task 2, depth perception was evaluated through a precise comparison between the panoramas and the relative abstract models. Participants selectively focused on distance to the target wall or the size of the target wall in the front view, ignoring the left and right monitors. However, in stimulus group D, as broader information appeared, participants’ visual attention was possibly affected by the perspective of the interior space, turning from the front view to the side view, which diverted participants’ attention to the farther area of the garden.

### Comparison of methodologies for measuring distance perception

Depth perception was more strongly correlated with a wider range of variables in Task 1. This indicates the potential influence of methodology (i.e., experimental devices and perceptual measurement methods) on distance perception estimation. Many studies explore the differences between measurement methods (Messing and Durgin, 2005; Peer and Ponto, 2017; Maruhn et al., 2019). The present study shows that the immersive virtual circumstance is superior in creating an approximate sensation of the real environment compared to a traditional display. However, the deficiency here is that Task 2 was not carried out in the CAVE. Future research should further develop the interaction between participants and the virtual reality environment. The advantage of verbal estimation in examining the impact of varying visual variables on subjective perception should also be examined.

## Conclusion and future work

Aspect changes at every turn are a typical phenomenon in classical Chinese gardens. We quantitatively demonstrated this in terms of close interval observation points, providing a foundation to study the mechanism of how to obtain rich experiences through enhancement of perceptual variation and bias in a limited area. Furthermore, we showed the possible impact of ambient information provided by panoramic images on cognitive behavior.

Although the present system of simulation using panorama pictures cannot include the effects of motion parallax caused by head movement, we could overcome this limitation by using a 3D model presented on a virtual display to simulate the real environment in the future study.

Other directions for future research include taking advantage of panoramic properties to study physical environments, such as vision in motion (optical flow, motion parallax, optical occlusion, and disocclusion; Gibson, 2014). Moreover, dynamic rather than static displays (e.g., panoramic videos and detailed virtual models along paths) can be used (Heft and Nasar, 2000), combined with human behavior research (eye movement, walking and pausing, standing, and sitting).

## Data availability statement

The raw data supporting the conclusions of this article will be made available by the authors, without undue reservation.

## Ethics statement

The studies involving human participants were reviewed and approved by the Swiss Federal Institute of Technology Zurich Ethics Committee. Written informed consent to participate in this study was provided by the participants. The individual(s) provided their written informed consent for the publication of any identifiable images or data presented in this article.

## Author contributions

HZ and ZG: conceptualization. HZ, ZG, and RO: experimental design and data analysis. HZ: original draft and conducting experiment. YK and RO: supervision. All authors contributed to the article and approved the submitted version.

## Funding

This research was supported by the National Natural Science Foundation of China (grant nos. 52038007 and 51808094).

## Conflict of interest

The authors declare that the research was conducted in the absence of any commercial or financial relationships that could be construed as a potential conflict of interest.

## Publisher’s note

All claims expressed in this article are solely those of the authors and do not necessarily represent those of their affiliated organizations, or those of the publisher, the editors and the reviewers. Any product that may be evaluated in this article, or claim that may be made by its manufacturer, is not guaranteed or endorsed by the publisher.
